# Sterilization in Dental Practice

**Published:** 1912-04-15

**Authors:** L. E. Custer

**Affiliations:** Dayton, Ohio


### STERILIZATION IN DENTAL PRACTICE.

BY L. E. CUSTER, B.S., D.D.S., DAYTON, OHIO.

The public is today fast awakening to the fact that contagion lurks everywhere. There are on every hand disease-producing germs which are only waiting for a suitable field for development. Langfield says: "The germ of pneumonia is a constant inhabitant of almost every one's mouth, leading there a harmless parasitic existence; yet let the vital powers be reduced through fatigue, exposure or cold and it becomes an infectious agent." The wave of hygiene which is passing over our country and which is reaching all departments of activity will be productive of more good than any measure proposed in years.

I am not calling your attention to sterilization because the public demands it, but because I believe we have not been as careful in this respect in the past as we should have been.

A Chicago physician once said to me, "You dentists do not sterilize." He meant by this that we are not paying as close attention to it as does the physician and surgeon. We have been sterilizing, else how could we prepare and fill an infected pulp canal? But there are other fields demanding sterilization in dental practice; the operator's body and hands, the instruments which he uses and the field of operation. As cited in the beginning, there are infectious agents on every hand which are only waiting for favorable conditions for growth. There are a great many ways in which the dentist can open the tissues to infection, and I do not doubt that were it not for the natural slightly germicidal property of the saliva that infection would follow many dental operations. The naturally quick-healing property of the oral tissues, combined with the washings of the saliva, have prevented many an infection. The dog licks its wounds as if by instinct to prevent infection.

There is scarcely an operation made by the dentist but opens oral tissues. If it is not thus there may already be openings in the form of abrasions or the cracked corner of the mouth so often seen. The rubber dam may be applied, but even the act of applying, the pushing of a ligature under the gum or the application of a clamp may bring blood. The injection of an anesthetic, an extraction and the dressing of a tooth for a crown are especially dangerous operations.

The dentist by the very nature of his calling occupies a position which, if only for the sense of cleanliness, can well afford to pay closer attention to sterilization. Everybody thinks his mouth is the cleanest part of him and nothing shall enter it but the cleanest of fingers and instruments. But as a matter of fact, the mouth is a hot-bed of micro-organisms, and no cavity of the body presents such favorable conditions for their growth and development. Therefore, the dentist should for two reasons observe the closest details of sterilization. In one of the most recent surgical operations so much care is given to sterilization that the hands of the operator, no matter how clean and sterile they may be, do not touch the field of operation—all work is done with instruments which have been absolutely sterilized.

The first step should be the preparation of one's hands. It is not allowable for the operator to go from one patient to another without thoroughly washing his hands, and it is not sufficient to simply wash with water. Soap should be generously used. For my own use I have arranged a faucet of flowing warm water, which is operated by stepping on a little button protruding through the floor. Three things are gained by this—the hands are washed and rinsed by flowing water, which is preferable to a bowl of water; they do not touch a faucet handle, and they are warmed. A dentist should not touch a patient's face with cold hands. The tincture of greensoap serves not only as a soap but, for reasons shown later, is one of the best sterilizing agents for the hands.

During the operations we often do many things thoughtlessly which should not be done without again washing the hands. Perhaps the worst practice is the handling of money during an operation without washing, and even the opening of a door with the hand could be criticized. You might a few moments before have opened the door with hands stained with blood or pus. Watch the surgeon at the hospital and see how careful he is to touch nothing but sterilized objects.

The sterilization of the field of operation—the mouth—cannot be as thoroughly done as other parts of the body, but that does not excuse us from making the attempt. All operations upon the soft tissues should be preceded by thorough washing of the mouth with a germicide. The best for this will be touched upon later.

The sterilization of instruments is of prime importance, particularly all cutting instruments. The burs, by reason of the fine leaves and difficulty of cleansing, are especially dangerous. Thorough washing with flowing water should be the first step, and the next is the actual sterilization. The best agents today are surprisingly simple. Moist heat of boiling water or, still better, a steam boiler capable of twenty pounds' pressure is the surest of all agents. We should bear in mind that many of the most dangerous germs are encapsuled and nothing but heat will penetrate the envelope. The medicinal agents used for this purpose have been entirely revolutionized of late. My attention was called to this by a physician of this city and later on it appeared in *The Dental Summary*. Doctors Post and Nicoll of Rush Medical College made an exhaustive course of experiments with the common germicides, with the most surprising results. It was found that the germicides upon which we pinned our faith were less effective than other agents which were supposed to be less potent. For instance, bichloride of mercury, which has been the king of germicides for about thirty years, was found, even in the strongest solution allowable to use, to be less effective than common alcohol. It has been found that to be effective as a germicide it should corrode any steel instrument. If a weak solution, the instrument had to be immersed in it so long to be completely sterilized that it would be attacked by the agent.

Not only was the bichloride found to be surprisingly ineffective, but other agents which had stood high were found to be equally so. Incidentally, however, the surgeon has been washing his hands with the tincture of green soap, and we as dentists have been using Listerine in the mouth, little thinking that the alcohol contained in them was responsible for the success that was given to some other supposed-to-be sterilizer. It now appears that the agent which was the real sterilizing ingredient in the soap and in Listerine was the alcohol. I understand that the proprietary preparation known as Listerine is largely alcohol flavored with some of the essential oils. We have been drying out cavities and pulp canals with alcohol for its dehydrating qualities, little thinking that it possessed a strong germicidal property. It is to be regretted that in these tests the essential oils were not also tried out. We have been taught that the oil of Cassia, the most powerful oil sterilizer, is equal to the bichloride of mercury as a sterilizing agent.

These tests showed, in summing up, that the silver and iodine preparations were exceedingly high in germicidal property, but the most surprising of all was the high germicidal property of alcohol. Indeed, it ranks first as a sterilizing agent. All germs are killed in less than a minute in even a 50 per cent. solution. How fortunate it is for the dentist that this is so, for it possesses properties which make it especially desirable. Besides its sterilizing properties, it is a dehydrating agent and does not injure the most delicate instruments. The mouth mirror may be immersed in it for days without being affected. The only objection, and this is trifling, is the dull brownish cast it gives to rubber-handled instruments when left in it for an excessive length of time. Drying of the instruments with a napkin is made quite unnecessary by reason of its high volatile property.

To make this practical, the hands should be washed with green soap, which contains a large percentage of alcohol, and more alcohol can be added if necessary. We today have very convenient appliances for dispensing liquid soap, which nicely serves this purpose.

The patient may in a measure sterilize the mouth with a 25 per cent. alcohol. If objection is made to alcohol use Listerine or any of the proprietary mouth washes which are known to contain a good percentage of alcohol.

For the sterilization of the instruments a developing tray such as is used by photographers, which has been ground true on its edges and covered with a piece of plate glass to prevent evaporation, serves very nicely. For all operations in the mouth pure grain alcohol should be used. This is rather too expensive to use in the sterilization of the instruments, but what is known as denatured alcohol, costing sixty cents per gallon, serves just as well. Denatured alcohol is about 90 per cent. alcohol, the other 10 per cent. is naphtha, or gasoline, which prevents its use for drinking purposes, but does not in any way injure it for use in the arts. In a late German article it is highly recommended for instrument sterilization.

In conclusion, let us pay a closer attention to sterilization, which is quite necessary in this day, thereby increasing the respect of our patients and refuting the charge of the Chicago physician, for in so doing we are preventing infection. A clean office, clean body and hands and sterile instruments are demanded today, and we honor ourselves and the profession by practicing sterilization in all its details.—*Summary.*

